# Continuous farmyard manure application increases nitrogen fixation capacity of soils and relative abundance of iron-oxidizing diazotrophs in nutrient-deficient paddy fields in Madagascar

**DOI:** 10.5511/plantbiotechnology.25.0414b

**Published:** 2025-09-25

**Authors:** Takanori Okamoto, Papa Saliou Sarr, Hidetoshi Asai, Yasuhiro Tsujimoto, Tomohiro Nishigaki, Toshiyuki Takai, Tantely Vahatra Rakotonindrina, Hobimiarantsoa Rakotonindrina, Andry Andriamananjara, Arisa Nishihara, Moriya Ohkuma, Motohiko Kondo

**Affiliations:** 1 Japan International Research Center for Agricultural Sciences (JIRCAS), Tsukuba, Ibaraki 305-8686, Japan; 2 Graduate School of Bioagricultural Sciences, Nagoya University, Nagoya, Aichi 464-8601, Japan; 3 Laboratoire des Radio-Isotopes (LRI), Université d’Antananarivo, 101 Antananarivo, Madagascar; 4 Japan Collection of Microorganisms (JCM), RIKEN BioResource Research Center, Tsukuba, Ibaraki 305-0074, Japan

**Keywords:** farmyard manure, iron-oxidizing bacteria, Madagascar, nitrogen fixation, rhizosphere

## Abstract

Farmyard manure (FYM)—a mixture of animal excreta and plant residues—remains an important nutrient resource for lowland rice production in sub-Saharan Africa (SSA). However, the underlying mechanism of FYM in altering microbiological communities and supplying nutrients for rice production remains poorly understood. Hence, this study aimed to elucidate the effects of FYM application on N fixation and the diazotroph communities in the nutrient-deficient lowlands of SSA. Soil samples from three farmers’ fields in Madagascar were incubated with ^15^N_2_ gas for 1 month to assess their N fixation capacity. The plots in each field were subjected to the following fertilizer conditions: control (no fertilizer inputs) or continuous application of FYM or mineral fertilizer for previous 4 years. One field with a high N fixation capacity was selected to analyze the nitrogenase (*nifH*) and 16S rRNA genes in the rhizosphere. The continuous application of FYM significantly increased the N-fixation capacity of the soil compared with one-time or no FYM applications, particularly in fields where FYM was highly effective on rice yield improvement. Continuous FYM application also significantly increased the relative abundance of *nifH* gene sequences close to iron-oxidizing bacteria (Family *Gallionellaceae*) in the rhizosphere, whereas this change did not occur with the continuous application of mineral fertilizer. These results imply that continuous FYM application enhances N fixation capacity via changes in the diazotroph communities. The high abundance of diazotrophs related to the oxidation-reduction process of iron may be associated with the iron-rich soils in the region.

## Introduction

Nitrogen (N) and phosphorus (P) deficiencies are major yield-limiting factors for lowland rice production in sub-Saharan Africa (SSA) (Johnson et al. 2023; Saito et al. 2019). In addition to the highly weathered and nutrient-poor soils that prevail in SSA, the majority of smallholder farmers have limited access to mineral fertilizer inputs physically and economically (Chianu et al. 2012; Rakotoson et al. 2022; Tsujimoto et al. 2019). In SSA, only 9.2 and 1.5 kg ha^−1^ of N and P fertilizer inputs, respectively, are allotted for agricultural production, which are far below the mineral fertilizer inputs in other countries (Rakotoson et al. 2022).

Consequently, locally available organic fertilizers such as farmyard manure (FYM)—a mixture of animal excreta, crop residues, and fodder that are piled nearby homesteads—remain an important nutrient resource for rice production. For instance, Ozaki and Sakurai (2020) surveyed 1,736 plots in the central highlands of Madagascar and reported that organic fertilizers were applied in 24% of lowland rice fields, while mineral fertilizers were only applied in 5% of lowland rice fields. Another study involving 357 rice farmers in Ethiopia, Madagascar, Rwanda, Tanzania, and Uganda revealed that 48% of farmers apply organic fertilizers for lowland and upland rice production (Senthilkumar et al. 2020). Therefore, organic fertilizer application for rice production remains an important practice in SSA, given the relatively low utilization of mineral fertilizer in the region.

However, further studies should be conducted to understand the effects and mechanisms of action such organic amendments for supplying N and P to the soil and for increasing rice yield in nutrient-deficient lowlands in SSA. As organic materials contain nutrients and carbon (C), their application affects nutrient dynamics in paddy soils via chemical and microbiological reactions.

In general, flooded conditions in paddy fields increase the soluble P content of soils via microbial reduction and increase in pH levels (Kirk 2004; Liptzin and Silver 2009; Rabeharisoa et al. 2012). Under flooded conditions, fresh organic inputs accelerate P solubilization by acting as electron donors, which intensify the microbial reductive dissolution of P-bearing iron (Fe) oxides (Rakotoson et al. 2015). Some studies have indicated that the reduction of P-bearing Fe oxides is carried out by Fe-reducing bacteria (Wang, Ma et al. 2021; Wang et al. 2022). Consequently, the effect of FYM application is significant for increasing rice yield in the Fe-rich and P-deficient lowlands in SSA (Asai et al. 2021; Rinasoa et al. 2023).

Some studies have reported that organic amendments such as rice straw enhance N fixation in paddy fields in Asian countries (Kondo and Yasuda 2003; Rao 1978; Tanaka et al. 2006). In rice cultivation systems, the amount of N fixation per crop season is estimated to be approximately 3–63 N kg ha^−1^ (Yoshida and Ancajas 1973). Charyulu et al. (1981) reported that the N fixation rate in rice straw-amended soil (6 t ha^−1^) increased by 32% compared with that in the control. Kondo and Yasuda (2003) also demonstrated that the long-term application of FYM and rice straw increased the potential for N fixation in paddy soils. In contrast, Tanaka et al. (2006) revealed that the addition of N-rich compost suppressed N fixation by 50%. These contradictory results are partly attributed to differences in the chemical and biological properties of soils, including the composition of diazotroph communities. Organic amendments have been suggested to affect heterotrophic diazotrophs (Kondo and Yasuda 2003; Zhang et al. 2022); however, their effects on specific diazotroph communities remain unclear. N fixation is an energy-intensive process, and diazotrophs consume large amounts of P and carbon sources (Olivares et al. 2013). Therefore, the impact of organic amendments on rice production can be enhanced in Fe-rich and P-deficient lowlands, where these amendments first supply P and C sources via the aforementioned processes, thereby activating N fixation by diazotrophs. However, no studies have been conducted to determine how FYM application affects N fixation and diazotroph communities in paddy soils, especially in the rhizosphere, which is known to be a hotspot for N fixation where exudates and O_2_ are supplied from the roots (Charyulu et al. 1981; Yoneyama et al. 2017).

Hence, the aim of this study was to elucidate the effects of FYM application on N fixation by conducting a microcosm soil incubation experiment using soil samples that had been enriched with FYM for 4 consecutive years in Fe-rich rice paddy soils in Madagascar, a typical example of P-deficient soil in SSA. Moreover, the major diazotroph community structures that contribute to N fixation in the rhizosphere soil of rice plants were identified in a field that showed the greatest increase in both grain yield and aboveground N uptake at maturity due to FYM application. The effects of FYM application on those communities were then compared with those of inorganic N and P fertilizers.

## Materials and methods

### Aerobic soil incubation with ^15^N_2_ gas in microcosm

Consecutive fertilizer management trials by combining FYM and mineral fertilizer were implemented for over 4 years in three farmers’ fields (F1, F2, and F3) in the communes of Behenjy (19° 10′ S, 47° 29′ E) and Antohobe (19° 46′ S, 46° 41′E), which are both located in the central highlands of Madagascar. After the harvest of rice at the end of the 4th year, when the fields were no longer flooded, soil samples for incubation tests were collected at a depth of 0–15 cm in plots subject to two different fertilizer conditions: the “poor soil” plot, where rice was continuously grown without mineral or organic fertilizer inputs for 4 years, and the “enriched soil” plot, where rice was grown without mineral fertilizer inputs but with continuous FYM application at a rate of 8 t ha^−1^ year^−1^ for 4 years. Thus, two soil types—poor and enriched—in each experimental field (F1, F2, and F3) were incubated. Fields F2 and F3 were highly responsive to FYM application, while F1 was less responsive to FYM treatment (Asai et al. unpublished). The properties of the soil samples are listed in Table 1. Total C, total N, and oxalate-extractable P were higher in the enriched soils than in the poor soils in all fields. Easily decomposable organic C was higher in enriched soils than in poor soils in F2 and F3. Free Fe concentrations ranged from 10 to 19 mg kg^−1^. F1 had relatively high oxalate-extractable P and low free Fe content.

**Table 1:** Table 1. Soil properties in three paddy fields in the central highlands of Madagascar.

	F1		F2		F3	
	Poor soil	Enriched soil	Poor soil	Enriched soil	Poor soil	Enriched soil
pH	5.5	5.4	5.6	5.5	5.2	5.1
Total C (%)	1.1	1.2	1.3	1.5	1.1	1.3
Total N (%)	0.10	0.10	0.11	0.13	0.11	0.12
Easily decomposable organic C (mg kg^−1^)	474	465	456	548	502	529
Oxalate-extractable P (mg kg^−1^)	159	171	65	85	77	81
Free Fe (g kg^−1^)	11	9	15	17	19	19

“Poor soil” plot where rice was continuously grown without mineral or organic fertilizer inputs for four years; “Enriched soil” plot where rice was grown with continuous FYM application. pH was determined in a 1 : 2.5 ratio of soil:water; total C and N were determined using the combustion method with an organic elemental analyzer (Sumigraph NC220F; Sumika, Tokyo, Japan); Easily decomposable organic C by measuring dissolved organic C extracted from oven-dried soils shaken with distilled water (Azuma et al. 2015); Oxalate-extractable P by the acid ammonium oxalate method (Courchesne and Turmel 2008); Free Fe by the citrate-dithionite method (Holmgren 1967; Mehra and Jackson 1958).

Two incubation treatments in three replicates, −FYM and +FYM, were applied for each soil type. In both treatments, 2 g of soil was sifted through a 2-mm sieve, air-dried at room temperature, and were put in 20-ml vials (SVG-20; NICHIDEN-RIKA GLASS, Hyogo, Japan). In the +FYM treatment, 8 mg of FYM locally produced in Madagascar (0.4% soil), consisting of 13% C, 0.98% N, and 0.3% P, was mixed with the soil samples stored in the vials. The amounts of C, N, and P added by the FYM application were 1, 0.078, and 0.024 mg per vial, respectively. FYM was air-dried and pulverized prior to application.

After the soil samples and FYM were combined with 5 ml of distilled water, the vials were placed on butyl rubber stoppers and vacuumed using a degasser (EN-1004; Endo-Rika, Hokkaido, Japan). Afterward, the vials were filled with 80% N_2_ gas (25 atom% ^15^N; Shoko Science, Kanagawa, Japan) and 20% oxygen gas. The vials were incubated under 14 h of light and 10 h of dark conditions at 25°C (MLR-350T; SANYO Electric, Osaka, Japan). After 30 days of incubation, the vials were opened, and the soil samples were dried at 80°C. Subsequently, the soil samples were ground and placed in tin capsules; their total N concentration and ^15^N ratio were determined by using the Flash2000-DELTAplus Advantage ConFloIII System (ThermoFisher Scientific, Waltham, MA, USA).

### Rhizosphere soil sampling and DNA extraction

Of the aforementioned three fields, F3 was selected for further analysis of the bacterial community composition in the rhizosphere of rice plants because it exhibited the greatest increase in both grain yield and aboveground N uptake at maturity due to FYM application. At the physiological maturity stage of the rice plants when the field was continuously flooded, one plant per plot was uprooted from different mineral fertilizer application treatments (control, N, P, and NP) in both “poor soil − FYM” plots (FYM was not applied for either the current season or preceding 3 years) and “enriched soil + FYM” plots (FYM was continuously applied for the current season and preceding 3 years). Afterward, the roots were gently shaken to remove soil residues that did not adhere to the root surface. The soil particles that tightly adhering to the root surface was carefully collected with brushes and labeled as rhizosphere soil (Yukun et al. 2021). Approximately 20 g per sample of the collected rhizosphere soil was placed in 50-ml tubes and stored at −20°C until DNA extraction. DNA extraction was performed using the FastDNA SPIN Kit for Soil (MP-Biomedicals, Irvine, CA, USA), according to the kit instructions. In the N, P, and NP plots, N fertilizer (urea, 80 kg-N ha^−1^ in total with 50 kg-N as basal and 30 kg-N as top dress), and P fertilizer (triple superphosphate, 22 kg-P ha^−1^ as basal application) were applied for 4 consecutive years.

### Quantitative PCR and amplicon sequencing

The abundance of the nitrogenase (*nifH*) gene was determined by performing quantitative PCR (qPCR), as following the methods described by Sarr et al. (2020), using the PolF/PolR forward (5′-TGCGAYCCSAARGCBGACTC-3′) and reverse (5′-ATSGCCATCATYTCRCCGGA-3′) primers (Poly et al. 2001). qPCR was performed using a 15-µl reaction mixture composed of the following: 7.5 µl of SsoAdvanced Universal SYBR Green Supermix (1725271; BioRad Laboratories, Hercules, CA, USA), 0.0375 µl of 100 µM of each primer, 1 µl of one-tenth diluted DNA, and 6.425 µl of sterilized Milli-Q water. qPCR reactions were run on the CFX96 Touch Real-Time PCR Detection System (Bio-Rad Laboratories). Before the qPCR reactions, a plasmid standard was generated by cloning a *nifH*-amplified PCR product using the PolF/PolR primers. The procedures for cloning, plasmid extraction, and quantification of the initial cell quantity are described in Sarr et al. (2020). The optimal annealing temperature for the qPCR of *nifH* was determined by a gradient qPCR and was set to 57°C. The amplification efficiency ranged from 88% to 100%, and the R^2^ values were between 0.997 and 0.998.

Amplicon sequencing was performed by the Bioengineering Lab (Kanagawa, Japan). The amplicon PCR reaction for the *nifH* and V4 region of the 16S rRNA gene was conducted using the PolF/PolR (Poly et al. 2001) and 515F/806RB (Apprill et al. 2015) primers, respectively. Index PCR was performed again following the manufacturer’s instructions (16S Metagenomics Sequencing Library Preparation; Illumina, San Diego, CA, USA). Sequencing was conducted on the Illumina MiSeq system using a MiSeq Reagent Kit v3 (Illumina Inc.) with a 2×300 cycle configuration. Sequences with a quality value below 20 were removed using a Sickle (ver. 1.33) (Joshi and Fass 2011). The paired-end read-binding script, FLASH (ver. 1.2.11) (Magoč and Salzberg 2011) was used to bind the reads with a minimum overlap of 10 bp. After removing chimeric and noise sequences, representative sequences [amplicon sequence variants (ASVs)] were generated using the ASV method with the dada2 plugin in Qiime2 (ver. 2021.11) (Bolyen et al. 2019). The taxonomic classification of ASVs of the 16S rRNA gene were estimated using the feature-classifier plugin in Qiime2 against the Greengene database (ver. 13_8). For the *nifH* gene amplicon data, ASV sequences were translated into amino acid sequences, and sequences without Cys 97 and Cys 132 amino acids (protein numbering for the NifH protein in *Azotobacter vinelandii*; and 4Fe-4S iron-sulfur cluster ligating cysteines) (Howard et al. 1989) were removed. The top 30 ASVs, ranked by mean relative abundance across treatments, were estimated by comparing the NCBI Protein BLAST (blastp) searches against the non-redundant protein sequence (nr) database, excluding uncultured bacteria, to verify the taxonomic affiliations. The remaining ASVs were searched using Protein-Protein NCBI BLAST+ 2.6.0 against the NifH database named “NifH_AN2023_DB” (Supplementary Dataset1) we created by collecting the *nifH* sequences as follows. All NifH homologs were collected from protein_faa_reps in the genome taxonomy database (GTDB r214) (Parks et al. 2022) based on homology searches (mmseqs2 [ver. 13.45111 (ref1)]) (Steinegger and Söding 2018) using biochemically characterized proteins curated in SwissProtKB (ref2) (Boutet et al. 2007). Sequences that retained conserved cysteine-containing motifs such as the MgATP binding site ('YGKGGGIGK') and 4Fe-4S ligands ('PGVGC' and 'VVCGG') were extracted and used for the database. NifH clusters were assigned based on phylogenetic analysis and according to the classification criteria of Boyd et al. (2011) and Zehr et al. (2003).

A phylogenetic tree of *nifH* was constructed using each of the top 15 ASVs. These ASVs, along with reference sequences obtained via blastp or blantn, were aligned using the CLUSTAL W software (Larkin et al. 2007), with the default settings of MEGAX (Kumar et al. 2018). Phylogenetic trees were constructed using the maximum likelihood method by adopting the LG+G model (Le and Gascuel 2008). The robustness of the tree topologies was tested using 1,000 bootstrap replicates. *Roseiflexus castenholzii* DSM 13941 (GCA 000017805.1) was designated as the outgroup. For the reference strains used in the phylogenetic trees, N fixation, Fe oxidation, and Fe reduction abilities were estimated at the genus level using available information. Species names were according to GTDB r214. Then, the ASVs of the *nifH* gene were classified into NifH clusters.

### Statistical analyses

Three-way analysis of variance (ANOVA) was performed using EZR (Saitama Medical Center, Jichi Medical University, Saitama, Japan) (Kanda 2013), which is a graphical user interface for R version 4.3.3 (The R Foundation for Statistical Computing, Vienna, Austria), to analyze the effects of field, soil type, and FYM application in each treatment in the microcosm experiment Tukey’s HSD test was used to compare treatment differences in the microcosm experiments using EZR. The Shannon index for each sample was calculated using the vegan package in R. Two-way ANOVA was also performed using EZR to examine the effects of FYM and chemical fertilizer application in each treatment on the amplicon analyses.

## Results

### ^15^N fixation rate in rice paddy soils in the microcosm experiment

**Figure 1:**
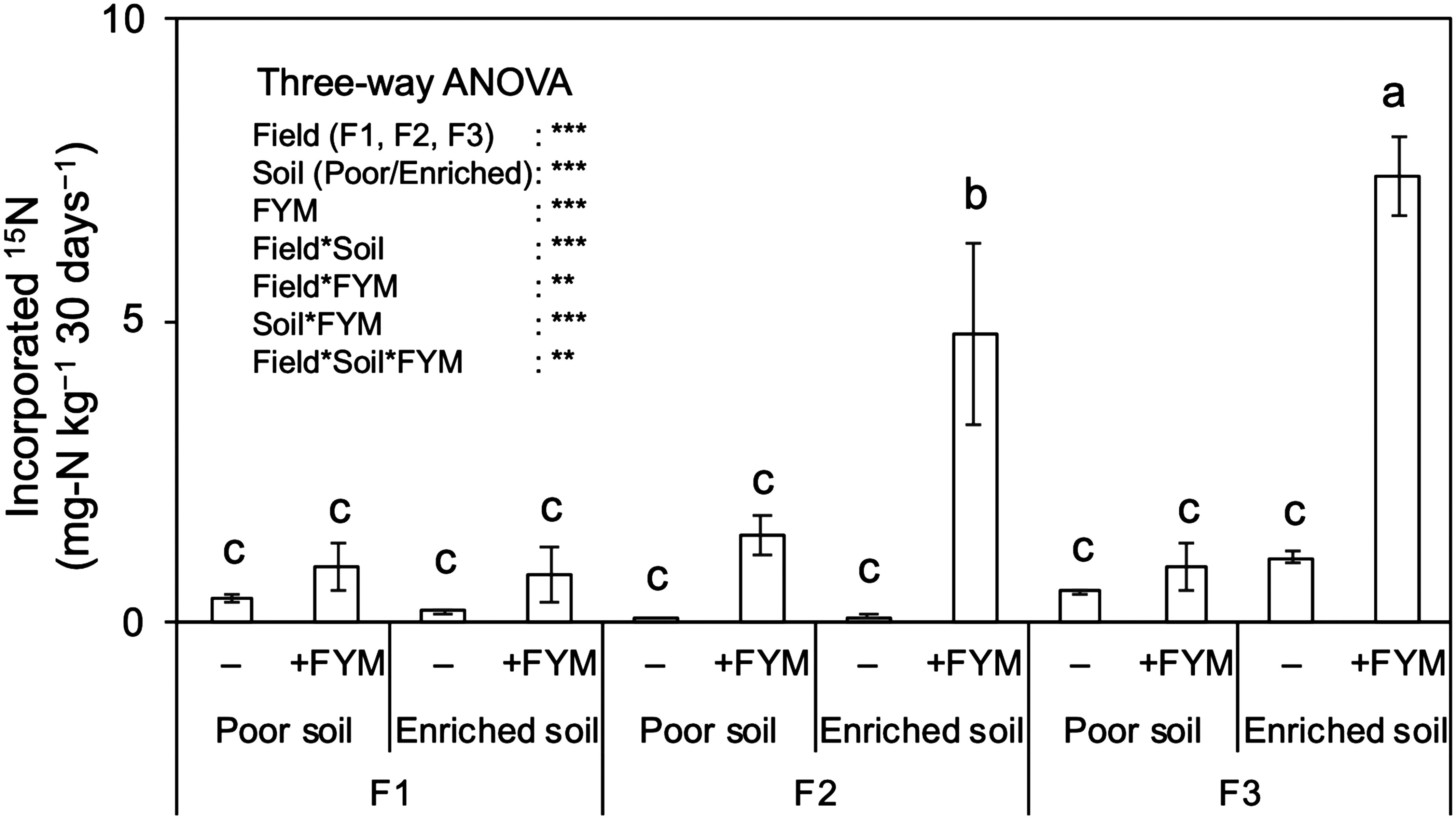
Figure 1. The amount of ^15^N incorporated in rice paddy soils with/without farmyard manure (FYM) added in a microcosm experiment. “Poor soil” plot where rice was continuously grown with no mineral or organic fertilizer inputs for four years; “Enriched soil” plot where rice was grown with continuous FYM application were used. Data are mean relative abundances±SE (*n*=3). *** and ** indicate significance at *p*<0.001, and 0.01, respectively. *p*-values are based on the results of three-way ANOVA. Different lowercase letters indicate significant differences based on Tukey’s HSD test (*p*<0.05).

As a result of soil incubation using ^15^N_2_ gas that was conducted to verify whether soil and FYM affect N fixation, the ^15^N atom% was higher than that in the soil before incubation in all the treatments, confirming that atmospheric ^15^N_2_ had been incorporated into the soil (0.04–7.42 mg-N kg^−1^ 30 days^−1^) (Figure 1). ANOVA results clearly demonstrated that the main effect of *Field*, *Soil* and *FYM* was significant (Figure 1). As for *FYM* factor, FYM-treated soils had significantly higher amounts of fixed ^15^N_2_ than those soils without FYM addition (2.73 vs 0.40 mg-N kg^−1^ 30 days^−1^ in average). Higher amounts of fixed ^15^N_2_ were also observed in Field 2 and Field 3 than Field 1 for Field factor (2.05 vs 0.59 mg-N kg^−1^ 30 days^−1^ in average) and in enriched soil than poor soil for Soil factor (2.50 vs 0.73 mg-N kg^−1^ 30 days^−1^ in average) (Figure 1). The results also showed that these three factors significantly interacted among all the treatments; ^15^N_2_ fixation rate was highest in the FYM-treated enriched soil in F3 (7.42 mg-N kg^−1^ 30 days^−1^; 8.7 times higher than the other treatments in F3), followed by the FYM-treated enriched soil of F2 (4.79 mg-N kg^−1^ 30 days^−1^; 8.8 times higher than the other treatments in F2) (Figure 1). The amount of fixed ^15^N_2_ in the FYM-treated enriched soil of F1 (0.80 mg-N kg^−1^ 30 days^−1^) was 1.5 times higher than that in the other treatment in F1 (Figure 1).

### Copy number, diversity, structure, and composition of the *nifH* and 16S rRNA genes

**Figure 2:**
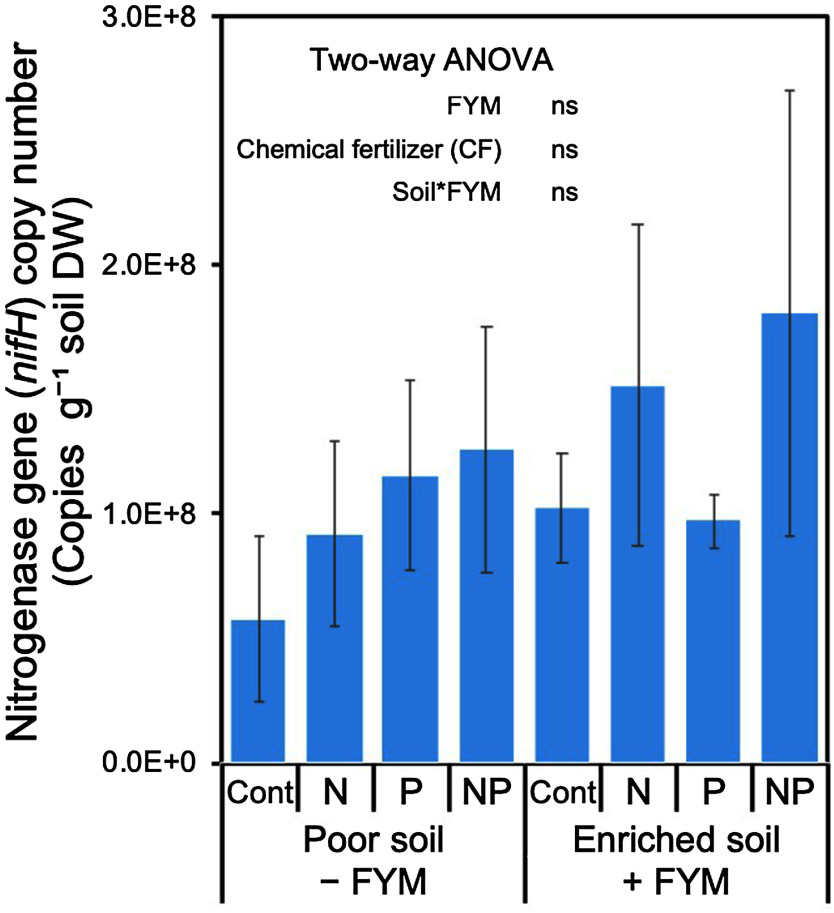
Figure 2. Quantification of the universal *nifH* gene in rice rhizosphere soils sampled from F3. “Poor soil − FYM” plot where rice was continuously grown without FYM application for current and past 3 years; “Enriched soil + FYM” plot where rice was grown with continuous FYM application for current and past 3 years were used. Data are means relative abundances±SE (*n*=3). “ns” indicates not significant. *p*-values are based on the results of two-way ANOVA.

As a result of qPCR analysis for DNA extracted from the rhizosphere soils during the rice cultivation in F3 to investigate the effects of FYM and chemical fertilizers on the amount of diazotrophs, no significant difference in the copy number of the universal *nifH* gene was detected in both FYM and chemical fertilizer treatments (Figure 2). The raw, valid reads, ASVs, and Shannon index data of the *nifH* and 16S rRNA genes obtained from the amplicon analysis are presented in Supplementary Table S1. The amplification of the *nifH* gene generated 21,241–41,952 valid reads and 922–1,369 ASVs from the 24 samples, while the amplification of the 16S rRNA gene yielded 29,228–39,468 valid reads and 734–1,225 ASVs. The Shannon index ranged from 5.7 to 6.6 for the *nifH* gene and 6.2 to 6.7 for the 16S rRNA gene, and FYM or chemical fertilizer application did not affect both genes (Supplementary Table S1).

**Figure 3:**
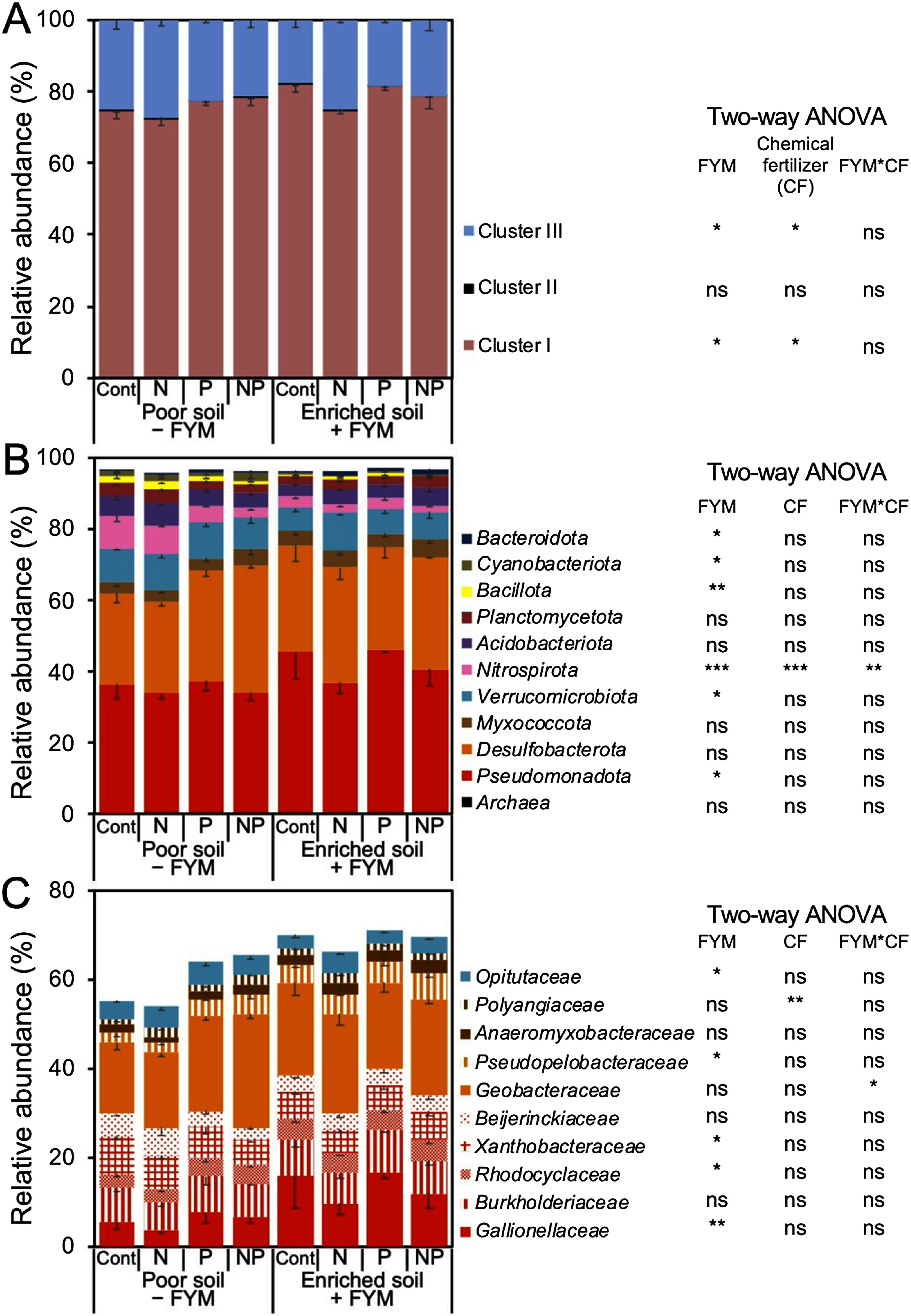
Figure 3. Comparisons of (A) NifH cluster, (B) phylum level (representative 10 bacterial phyla and domain *Archaea*), and (C) family level (representative 10 family) of relative abundance of *nifH* gene obtained from rice rhizosphere soils at F3. “Poor soil − FYM” plot where rice was grown without FYM application for current and past 3 years; “Enriched soil + FYM” plot where rice was grown with continuous FYM application for current and past 3 years were used. Data are means relative abundances−SE (*n*=3). ***, **, and * indicate significance at *p*<0.001, 0.01 and, 0.05, respectively. “ns” indicates not significant. *p*-values are based on the results of two-way ANOVA.

Based on the sequence analysis of the *nifH* gene, NifH cluster I was the most abundant (72.2–81.1% of the total reads) in all treatments, followed by cluster III (17.9–37.4%) and cluster II (0.2–0.5%); none was grouped into cluster IV (Figure 3A). At the domain level, most soil microorganisms were classified as bacteria (96.3–97.5%). At the phylum level, *Pseudomonadota* (33.5–45.5%) and *Desulfobacterota* (25.4–35.6%) were approximately equally abundant (Figure 3B). The phylum *Pseudomonadota*, *Desulfobacterota*, and *Myxococcota* were previously classified as phylum *Proteobacteria*, and their combined abundance ranged from 62.4% to 78.9% (Figure 3B) (Waite et al. 2020).

At the family level, *Geobacteraceae* (phylum *Desulfobacterota*) was the most abundant across all treatments (15.9–25.5%) (Figure 3C). The relative abundance of *Gallionellaceae* (phylum *Pseudomonadota*) increased with FYM application but not with mineral fertilizer treatments. *Gallionellaceae* was the second-most abundant bacterial family in the enriched soil+FYM treatment (9.7–16.5%) (Figure 3C). In the poor soil – FYM treatment, the second-most abundant bacterial families were any of *Beijerinckiaceae, Burkholderiaceae*, and *Xanthobacteraceae* (phylum *Pseudomonadota*) (Figure 3C).

Sequence analysis of the 16S rRNA gene revealed that at the domain level, the majority of sequences were classified as bacteria (97.6–98.5%) (Supplementary Figure S1). At the phylum level, *Acidobacteriota* had the highest relative abundance (19.8–22.7%) across treatments (Supplementary Figure S1), followed by *Pseudomonadota* (16.2–18.4%) and *Chloroflexota* (13.8–16.6%), with approximately equal relative abundance (Supplementary Figure S1). At the family level, *Koribacteraceae* (phylum *Acidobacteriota*) had the highest relative abundance (9.6–11.8%) in all treatments. The bacterial families with a relative abundance of 2% or more included *Bryobacteraceae* (2.5–3.1%; phylum *Acidobacteriota*), *Burkholderiaceae* (1.8–2.5%; phylum *Pseudomonadota*), *Anaerolineaceae* (2.4–3.4%; phylum *Chloroflexota*), *Ktedonobacteraceae* (1.9–3.4%; phylum *Chloroflexota*), *Anaeromyxobacteraceae* (2.7–5.2%; phylum *Myxococcota*), and *Geobacteraceae* (2.1–4.0%; phylum *Desulfobacterota*) (Supplementary Figure S1).

### Community analysis of potential diazotrophs and their Fe oxidation or Fe reduction capacity

**Figure 4:**
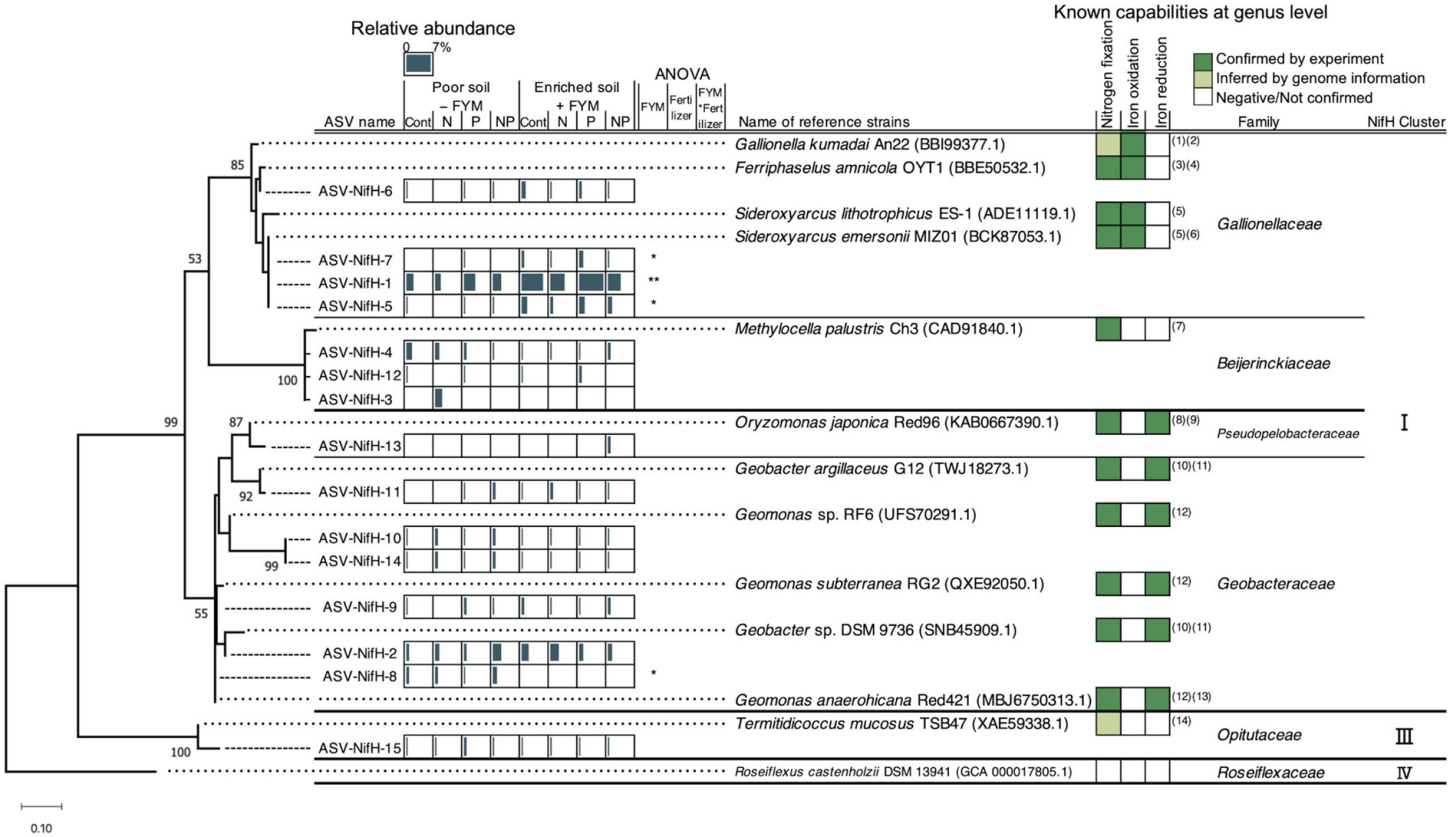
Figure 4. Maximum likelihood phylogenetic tree of NifH amplicon sequence variants (ASVs) obtained from rice rhizosphere soils at F3. “Poor soil−FYM” plot where rice was grown without FYM application for current and past 3 years; “Enriched soil + FYM” plot where rice was grown with continuous FYM application for current and past 3 years were used. Bootstrap values ≥50% are indicated at their respective nodes. The bar graphs on the right hand of the ASV-NifH name indicate the mean relative abundance of the ASVs (*n*=3). ** and * indicate significance at *p*<0.01 and 0.05, respectively. *p*-values are based on the results of two-way ANOVA. The green-and-white shading in the columns on the right hand of the reference strains are colored according to known capabilities of nitrogen fixation, iron oxidation, and iron reduction of those bacteria based on the data from (1) Watanabe et al. (2021); (2) Khalifa et al. (2018); (3) Kato et al. (2015); (4) Kato et al. (2013); (5) Emerson et al. (2013) ; (6) Kato et al. (2022); (7) Dedysh et al. (2000); (8) Masuda et al. (2024); (9) Xu et al. (2020); (10) Bazylinski et al. (2000); (11) Shelobolina et al. (2007); (12) Liu et al. (2022); (13) Zhang et al. (2021); (14) Mei et al. (2024).

Phylogenetic analysis was conducted using the top 15 ASVs with the highest relative abundance to clarify the characteristics of ASVs in more detail. The result showed that all the ASVs were highly identical to bacteria known or predicted to have an N-fixing ability (Figure 4). The top 14 ASVs were grouped into NifH cluster I (Figure 4). Among the fifteen ASVs, four were classified as Fe-oxidizing bacteria, three as methanotrophic bacteria, and seven as Fe-reducing bacteria (Figure 4). The top 30 ASVs of the *nifH* (ASV-NifH-1–30) and 16S rRNA genes (ASV-16S-1–30) are listed in Supplementary Tables S2 and S3, respectively.

ASV-NifH-1, classified as the family *Gallionellaceae*, was abundant in all treatments; however, its relative abundance increased in enriched soil+FYM but not in chemical fertilizer treatments (Figure 4, Supplementary Table S2). Sequences of ASV-NifH-1, -5, and -7 were 100% identical to *Sideroxyarcus emersonii* MIZ01 (BCK87053.1), which is known to have an Fe-oxidizing ability (Kato et al. 2022) (Figure 4). *Sideroxydans lithotrophicus* ES-1 (ADE11119.1) is known to have an N-fixing ability (Emerson et al. 2013) (Figure 4). ASV-NifH-6 showed 99.1% identity with *Ferriphaselus amnicola* OYT1 (BBE50532.1), an Fe-oxidizing diazotroph (Kato et al. 2013, 2015) (Figure 4). Within the family *Beijerinckiaceae*, ASV-NifH-3, -4, and -12 were 98.1% identical to *Methylocella palustris* Ch3 (CAD91840.1), known as an N-fixing methanotroph (Dedysh et al. 2000), and were abundant in the control and N treatments in poor soils (Figure 4).

Seven ASVs (ASV-NifH-2, -8, -9, -10, -11, -13, and -14) exhibited 91.6–100% identity to the reference strains of the genera *Geobacter*, *Geomonas*, and *Oryzomonas* (phylum *Desulfobacterota*), which are all Fe-reducing diazotrophs (Bazylinski et al. 2000; Liu et al. 2022; Masuda et al. 2024; Shelobolina et al. 2007; Xu et al. 2020; Zhang et al. 2021) and were present in all treatments at a relative abundance range of 2.9–6.1% (Figure 4). ASV-NifH-2, which had a high relative abundance (0.7–2.5%) among the seven ASVs, was 98.1% identical to *Geobacter* sp. DSM 9736 (SNB45909.1) (Figure 4).

In the 16S rRNA gene, ASV-16S-5 was 100% identical to *Sideroxyarcus* sp. PN022 (HQ117918.1), an Fe-oxidizing bacterium (Emerson et al. 2013), and its relative abundance increased in the enriched soil+FYM treatment (Supplementary Table S3). ASV-16S-3, -8, and -14 displayed 93.5–94.8% identity with Candidatus Koribacter versatilis Ellin345 (CP000360.1), which is predicted to have an Fe-oxidizing ability (Zhang et al. 2023) (Supplementary Table S3). ASV-16S-6 was 100% identical to a strain of *Sphingomonas* sp., that is known to have Fe-reducing ability (Ding et al. 2015) (Supplementary Table S3). ASV-16S-1 showed 88.1% identity with a strain of *Longilinea* sp. that is inferred to have Fe reducing ability (Liu et al. 2017) and a high relative abundance (1.0–1.8%) (Supplementary Table S3).

## Discussion

The incubation trial using ^15^N_2_ gas demonstrated that the N-fixation capacity of the FYM-treated soils significantly increased across a range of soil types. The degree of change was prominent when FYM was consecutively applied for 4 years (enriched soil vs. poor soil) and where the soils had low available P and high free Fe contents (F2 and F3 vs. F1). The amount of atmospheric ^15^N_2_ incorporated into the soil was 8.7 times greater in FYM-treated enriched soil than soils in the other treatments in the same F3 field. Bei et al. (2013) reported that the incubation of surface paddy soil for 70 days resulted in the fixation of 6.91 mg-N kg^−1^, roughly estimated at 24 kg-N ha^−1^. The N-fixation rate in the FYM-treated enriched soil in F3 was much greater (7.42 mg-N kg^−1^) during the 30-day incubation period, implying the impact of continuous FYM application to supply N via atmospheric fixation. More importantly, the differences in the amount of ^15^N in the incubated soils corresponded to the plant responses to FYM application in the field trials. Compared with –FYM treatment, continuous FYM application increased both grain yield and aboveground N uptake at maturity to a greater degree in F2 and F3 than in F1 (Asai et al. unpublished). In the 4th year when soil samples were collected, the FYM application increased grain yields by 1.4–2.2 t ha^−1^ and aboveground N uptake at maturity by 21–23 kg N ha^−1^ in F1 and F2 while these increases were merely 0.4 t ha^−1^ and 7 kg N ha^−1^ increase in F1 Therefore, the varying responses of lowland rice production to FYM application can be partly attributed to enhanced N fixation.

The accumulation of labile C in the soil samples combined with the presence of easily decomposable substrates—pulverized and powdered FYM—may explain why the N fixation capacity of soils was highly enhanced by continuous FYM application. Previous studies have shown that organic amendment increases N fixation capacity in lowlands by supplying substrate C (Kondo and Yasuda 2003; Rao 1978; Tanaka et al. 2006) whereas none of these studies demonstrated the impact of continuous application compared to one-time application. The labile C content of the soil generally accumulates with continuous organic amendments (Yu et al. 2020). The amount of easily decomposable organic C in F2 and F3 in this study increased by 5–20% with FYM applications (enriched soil vs. poor soil) for 4 consecutive years; whereas, enriched soil in F1 decreased by 2% compared to poor soil. Thus, the addition of fresh and easily decomposable substrates may have promoted microbiological activity and P cycling (Parham et al. 2002), especially in Fe-rich yet P-deficient soils. FYM with high P content made from pig manure was reported to be more effective at increasing lowland rice yield in the same study area (Rinasoa et al. 2023), implying the importance of readily available P for priming microbiological activities.

In the rhizosphere of a highly FYM-responsive field, the relative abundance of Fe-oxidizing diazotrophs, such as *Sideroxyarcus emersonii* MIZ01, significantly increased with continuous FYM application whereas no significant change was observed due to continuous mineral fertilizer application. This is consistent with an observation that the relative abundance of Fe-oxidizing bacteria increased by adding wheat straw to Fe-rich paddy soil (Wang, Ma et al. 2021). This change may be associated with the process by which FYM application, as a C source, induces soil reduction and increases the concentration of ferrous Fe in the rhizosphere. Previous studies have also reported that soil redox potential decreased and ferrous Fe concentrations increased when FYM was applied to paddy fields in the same study area (Rakotoson et al. 2015; Rinasoa et al. 2022). This group of Fe-oxidizing bacteria, including *Sideroxyarcus emersonii* MIZ01, grows microaerobically, chemolithoautotrophically and uses CO_2_ as a C source (Kato et al. 2022; Zhou et al. 2022), suggesting that FYM indirectly functions as a C source for Fe-oxidizing bacteria. Based on both *nifH* and 16S rRNA gene sequences, Fe-reducing bacteria belonging to the family *Geobacteraceae* were abundant in all treatments, consistent with the high abundance of Fe-reducing bacteria in paddy soils regardless of fertilization (Li et al. 2020; Masuda et al. 2023). Fe-reducing bacteria utilize acetate, a major metabolite of microbial rice straw decomposition, as an electron donor (Glissmann and Conrad 2000; Masuda et al. 2020). The C contained in FYM might have first been decomposed by heterotrophs, and then the Fe-reducing bacteria might have generated divalent Fe ions which was then used by the Fe-oxidizing bacteria. In contrast to the diazotrophs involved in the redox of iron, which has a large relative abundance, no clear effect of chemical fertilizers was detected. Some studies reported diazotroph community in submerged paddy soil was affected by the application of chemical fertilizers including P and N (Tan et al. 2003; Wang, Li et al. 2021). On the other hand, this study implied that the effect of FYM that contains C source was greater than the single P or N sources on the diazotroph community in the rhizosphere of rice plants on poor nutrient soils.

This study demonstrated that continuous FYM application increased the rate of N fixation and abundance of Fe-oxidizing diazotrophs in Fe-rich yet P-deficient paddy soils. However, in the present study, no direct observations were yet presented to indicate that the enhancement of N fixation capacity via FYM application was derived from the change in Fe-oxidizing diazotrophs or any other microbiological compositions in the rhizosphere. Further investigation is needed to demonstrate the direct relationship between diazotrophs and N fixation in rhizosphere particularly focusing on the O_2_ supply from plant roots that should support the inhabitation of Fe-oxidizing bacteria under flooded paddy soil culture. The specific component of FYM responsible for enhancing N fixation remains unclear. Rinasoa et al. (2022) demonstrated that FYM with a high P content and low C : P ratio was the most effective for rice production. Further research is needed to determine how C and P composition in FYM influences N fixation. Further research is also needed to determine the effects of high Fe oxide and low P in soils on N fixation. In addition, Fe-oxidizing bacteria, which have been shown to be affected by FYM, have been isolated from rice paddy soils (Khalifa et al. 2018) and nitrogenase genes (*nifD* and *nifK*) from the family *Gallionellaceae* have been reported to be detected more frequently in paddy soils than in upland soils (Masuda et al. 2024), suggesting that they are diazotrophs that are common in paddy soil. So far, however, Fe-oxidizing bacteria have focused on their ability to oxidize divalent Fe ions (Chan et al. 2023; Naruse et al. 2019; Watanabe et al. 2023) and have not been considered as key diazotrophs in paddy soils. It is necessary to clarify whether they actively contribute to N fixation in paddy fields. The family *Gallionellaceae* has been reported attracting attention in Fe-rich paddy fields in China and Burkina Faso (Wang, Ma et al. 2021; Watanabe et al. 2023). The high abundance of Fe-oxidizing bacteria may be linked to the high Fe oxide content in the soils, a common feature in SSA. Further studies are required to assess the prevalence of diazotrophs related to the oxidation-reduction process across different Fe-rich paddy fields on their role in N fixation.

## Abbreviations

ASV: amplicon sequence variant
FYM: farmyard manure
SSA: sub-Saharan Africa

## Accession numbers

Sequence information has been deposited in DDBJ/EMBL/GenBank under the BioProject accession number PRJDB19884, and BioSample accession numbers SAMD00857988–SAMD00858011. The raw sequences have also been deposited in the DDBJ Sequence Read Archive database under the accession numbers DRR627342–DRR627365 for the *nifH* gene sequences, and DRR627366–DRR627389 for the 16S rRNA gene sequences.

## Description of supplementary files

Supplementary Table S1. Reads, amplicon sequence variants (ASVs), and Shannon index for the *nifH* gene and 16S rRNA sequences obtained from rice rhizosphere soils in Madagascar.

Supplementary Table S2. Closest relatives, mean relative abundance, and analysis of variance (ANOVA) of top 30 *nifH* gene amplicon sequence variants (ASVs) of the maximum mean relative abundance among the treatment for each ASV.

Supplementary Table S3. Closest relatives, mean relative abundance, and analysis of variance (ANOVA) of top 30 16S rRNA gene amplicon sequence variants (ASVs) of the maximum mean relative abundance among the treatment for each ASV.

Supplementary Figure S1. Comparisons of phylum level (representative 10 bacterial phyla and domain *Archaea*), and family level (representative 10 family) of relative abundance of 16S rRNA gene obtained from rice rhizosphere soils.

Supplementary Dataset1. NifHDB_AN_2023.
