# Supplementary material for: Continuous farmyard manure application increases nitrogen fixation capacity of soils and relative abundance of iron-oxidizing diazotrophs in nutrient-deficient paddy fields in Madagascar
Source: Plant Biotechnol (Tokyo). 2025 Sep 25;42(3):345–55. doi: 10.5511/plantbiotechnology.25.0414b (PMC12573543; doi:10.5511/plantbiotechnology.25.0414b)
Supplement: Supplementary Data [file plantbiotechnology-42-3-25.0414b_s001.pdf]

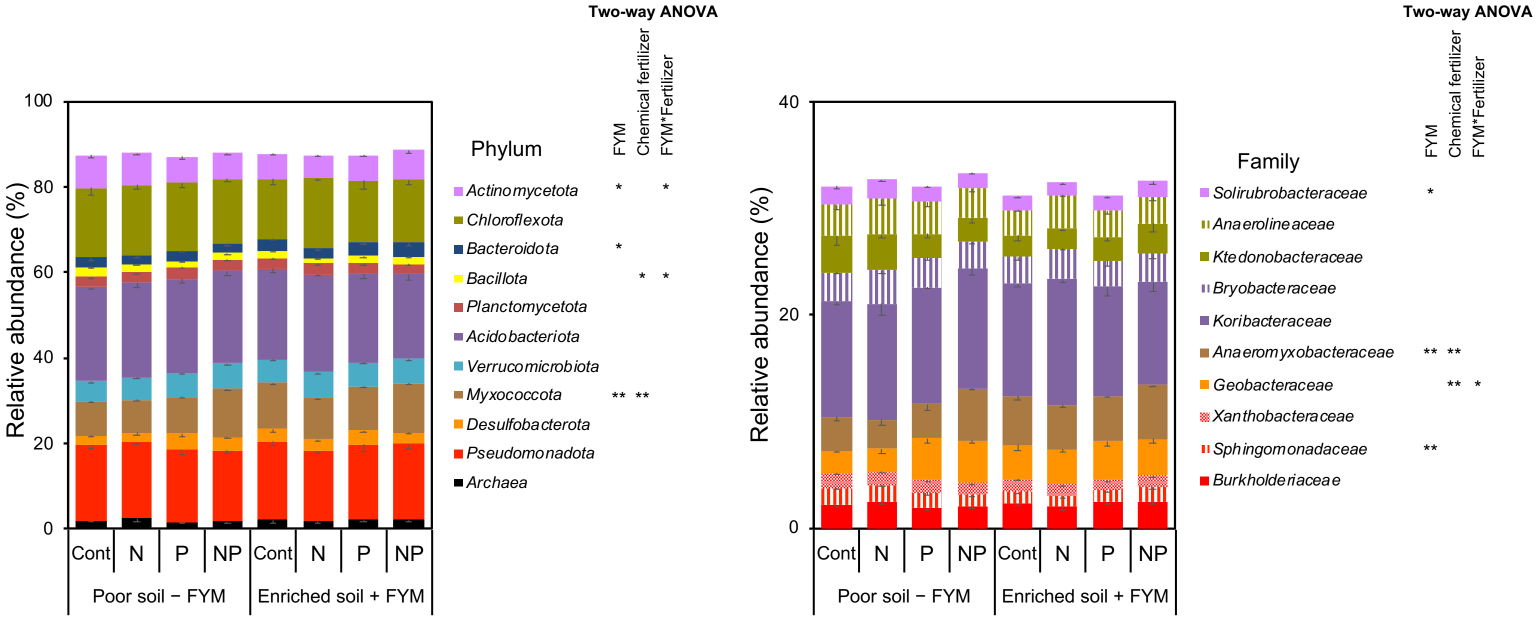

Supplementary Figure S1. Comparisons of phylum level (representative 10 bacterial phyla and domain *Archaea*), and family level (representative 10 family) of relative abundance of 16S rRNA gene obtained from rice rhizosphere soils. "Poor soil − FYM" plot where rice was grown without FYM application for current and past 3 years; "Enriched soil + FYM" plot where rice was grown with continuous FYM application for current and past 3 years were used. Data are means relative abundances − SE (n = 3).
